# A Clinical Case

**Published:** 1884-09

**Authors:** Charles T. Harris


### A CLINICAL CASE.

Case of Mrs. B., aged forty-nine years : Her ordinary weight, one hundred and seventy pounds ; height, five feet and eight inches, well proportioned; her temperament, nervo-lymphatic; subject to neuralgia.

When I was first called she was suffering from facial neuralgia and neuralgia fregeus. She was then relieved by Aconite, followed by Glonoine.

April 1st, 1883, and at a subsequent visit, I learned, on a more careful collation, that there was a dropsical diathesis, with the following history of the case: In 1860 she went with her husband as nurse into the United States service in the War of the Rebellion. She was then in robust health. In August of that year she contracted confluent variola of a very malignant type; her " limbs were badly swollen and looked like blood-blisters." She has never been well since. After returning home she was buried in the debris at the church disaster of the Central Baptist Church, eight years since. After that injury she was confined to her home for two weeks. She was generally bruised at that fall; heavy beams lay across her limbs; the limbs were hurt above the knees, where the timbers lay, leaving their impression on the limbs for days; the head was badly bruised and the whole body generally shocked and hurt from the injury. " Don't know if permanent bad effects followed the burial." She had dropsical œdema for about five years. One year ago in June acupuncture was resorted to three several times in different places, with discharge of a very considerable quantity of cold, clear, pellucid exudation, and Apis mel.[30] was exhibited, which lessened the œdema and enlargement of the limbs. Still there remained dispnœ, catching for breath, and gasping ; irregularity of the heart, dullness on percussion, and muffled beating of the heart. She had to *sit* in bed ; she had numbness in right arm and leg. In January last she was waked out of sleep with a "shock" and pain in head; her "head felt like a bladder of water swashed one way and another;" she had paralysis of the throat—could not *speak* or *swallow* any food or medicine. Gave Lachesis, mm potency, in disks under the tongue, and ordered food injections per rectum, which, I afterward learned, she did not take. She remained for three days without food or other medicine than that ordered. The second day gave Digitalis$^{2000}$, which aggravated very perceptibly. She had profuse discharge of water from the bowels and urinary organs, followed by gradual subsidence of the paralysis. But a severe headache intervened, which yielded to Belladonna. She continues the Dig. occasionally—weekly, if it does not aggravate too severely—but in minimum doses, and always in high potencies. The headache commonly yields to Bell.

The remedies used in her case were Apis mel., Lachesis, DIGITALIS, and Belladonna, as they were severally indicated. Her numbness and immobility of limbs gradually disappeared. She is now convalescing. She resumed her daily labor and is now housekeeping. Some numbness remains, at times, in right arm and side, for which I have ordered Rhus tox.

CHARLES T. HARRIS.

